# Varietal turn-over and their effect on yield and food security – Evidence from 20 years of household surveys in Kenya

**DOI:** 10.1016/j.gfs.2023.100676

**Published:** 2023-03

**Authors:** Hugo De Groote, Lumumba Brian Omondi

**Affiliations:** a International Maize and Wheat Improvement Centre (CIMMYT), Nairobi, Kenya; b College of Agriculture and Veterinary Sciences, University of Nairobi, Nairobi, Kenya

**Keywords:** Adoption, Maize, Kenya, Varietal turnover

## Abstract

Agricultural technology is key to food security in SSA, but new maize varieties are not able to replace the old, trusted ones. This study uses data from four representative household surveys conducted in Kenya over 21 years, to show that younger maize varieties have a clear, although limited, effect on yield (4 kg/ha/year controlling for fertilizer) and food security. Unfortunately, this is not sufficient to entice farmers to adopt them, and adoption rates have barely increased despite the market liberalization that brought many private seed companies and their varieties in the market, as the parastatal Kenya Seed Company continues to dominate the market. As a result, in combination with low fertilizer use, yields have stagnated for the last three decennia.

## Highlights

• This study uses data from 4 representative household surveys in Kenya over 21 years.
• Liberalization had only limited effect on private sector market share in maize seed.
• Varietal turnover only has a small effect on maize yield, compared to fertilizer.
• Few farmers adopt young varieties, adoption rates and yields hardly increased.
• Policies to increase private sector share, adoption of new varieties are presented.

## Graphical abstract

**Figure:**
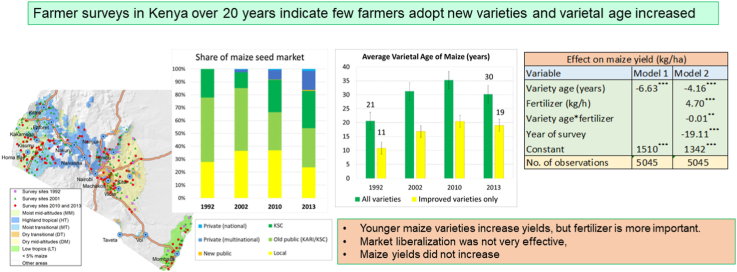

## 1 Introduction

Maize is the most important food staple in East and Southern Africa, but production is not keeping up with population growth (FAOSTAT, 2022). One of the main strategies to increase maize production and food security is the development and dissemination of improved maize varieties, used successfully in East and Southern Africa (De Groote et al., 2015; Smale and Thomas, 2003). However, while many new varieties have been released and disseminated, yield increases have been limited. One of the possible factors is the slow adoption of the latest varieties or low varietal turnover (Atlin et al., 2017; Nagarajan et al., 2019). Younger varieties perform substantially better in trials, with genetic gain for medium to late maturing varieties estimated at 79 kg ha^−1^ yr^−1^ under optimal conditions and 53 kg ha^−1^ yr^−1^ under low nitrogen conditions (Masuka et al., 2017). However, little is known about the effect of genetic gain of younger varieties in farmers’ fields. More generally, the effect of the adoption of improved maize varieties and, especially, varietal turnover on maize yields, maize production, and food security is not well understood. Therefore, in this paper, we analyze those relationships based on data from 20 years of representative household surveys in Kenya.

Agricultural intensification is the result of many factors, including population pressure (Boserup, 1965), particularly in relation to arable land (De Groote, 1999), scarcity or availability of resources (Ruttan and Hayami, 1984), investment in agricultural research (Evenson and Gollin, 2003), and policies (Spielman and Melinda Smale, 2017). In East and Southern Africa, the initial intensification of maize-based systems was driven by public research and extension systems (Smale and Thomas, 2003), but from the 1990s the emphasis has been on including the private sector through liberalization of agricultural markets (Jayne et al., 2002).

In Kenya, maize research started before independence and resulted in several popular high-yielding hybrids (Harrison, 1970). Seed production was commissioned to the Kenya Seed Company (KSC), originally a private seed company but later converted to a parastatal, and distribution to the Kenya Farmers’ Association (KFA), along with the accompanying fertilizer (Gerhart, 1975; Harrison, 1970). These efforts led to a substantial increase in maize yields in the 1960s and 1970s. The policy reforms of the 1990s aimed to reduce government expenses and making the markets more efficient though encouragement of private sector participation in agricultural input and output markets (Ariga et al., 2006; Wangia et al., 2004). Private seed companies entered the market and released many new maize varieties, but this did not lead to further yield increases, and yields stagnated from the 1980s till now (De Groote et al., 2005; Jena et al., 2020; Smale and Thomas, 2003).

Three major reasons for the stagnating yields can be considered: lack of progress in adopting improved technologies, lack of policies enabling that progress, and lack of a conducive environment. The two major technologies to be considered are low varietal turnover and low adoption of agronomic practices, especially soil fertility management. On varietal turnover, farmers in Kenya appreciate their old maize varieties and are not keen on moving on (Smale and Olwande, 2014), leading to slow varietal turnover (Naseem et al., 2018). Further, research and seed companies have not been particularly active in providing new varieties similar to those farmers like, especially late maturing varieties. As a result, average varietal age remains high (Abate et al., 2017; Naseem et al., 2018; Smale and Olwande, 2014). Second, Kenyan farmers have not been adopting the recommended agricultural practices to accompany improved varieties, in particular soil fertility management and fertilizer application (Jena et al., 2021). As has been observed in other SSA countries, the use of organic and chemical fertilizers is often too low to maintain soil fertility (Binswanger and Savastano, 2017).

In agricultural policies, three factors could impede technological progress. First, the liberalization was not fully achieved especially as, unlike other countries, the planned privatization of the national seed company KSC was never finalized, and it reversed to parastatal status in the 2013 restructuring (Executive office of the President, 2013). Factors other than profit-maximization enter the determination of KSC seed prices, which remain substantially lower than those from the competition. A court order also let KSC retain exclusive rights to many of the old, popular varieties (Naseem et al., 2018). Second, from 2000 onwards, policies emphasized the complementary roles for public and private sectors in ensuring efficient functioning of markets and resource allocation (MAFAP, 2013). This has led to a reduction of the role of the public sector, in particular in the extension and other support services for smallholders that could reduce the adoption of younger varieties (Doss et al., 2003), while the private sector has not fully filled the gap, leading to seed supply problems at the farm level (Smale and Thomas, 2003) and limited availability of improved hybrid seed (De Groote et al., 2005).

The changing conducive environment, finally, includes positive and negative factors. First, increased population density generally leads to population movement to urban areas, accompanied by increasing market participation for the remaining farmers (Binswanger and Savastano, 2017). It also brings an increased density of roads and markets, reducing the distance to the market and reduced transport costs. Both factors lead to increased intensification and an increase in the use of modern inputs such as improved varieties and fertilizer (De Groote, 1999). However, population density in Kenya remains very low and urbanization has just reached 27% (FAOSTAT, 2021), possibly too low to trigger increased market participation and intensification. On the negative side, reduced availability of land also leads to smaller farms and, in Kenya, the break-up of the large commercial farms taken over from European settlers. Smaller farms typically have less resources and therefore face more difficulties accessing modern inputs. Further, decreasing land availability pushes farmers into more marginal areas, less suitable for maize and for the use of modern inputs, leading to lower intensification and yield (Masters, 2013).

To better understand the factors that affect the stagnating maize yields in Kenya, we analyze the trends in adoption of improved maize varieties and in varietal turnover over a period of two decades, using a unique data set combining four different household surveys. We further analyze if the liberalization of agricultural markets led to an increased market share of the private seed sector, and test if, as argued, varietal turnover increases yields and food security.

The specific objectives of this paper are therefore to i) analyze the trends and factors in the adoption of improved maize varieties over the last 20 years; ii) analyze the trend in private sector participation in the seed market over time; iii) analyze the trends and factors in varietal turnover over time; iv) examine the effect of varietal age, in combination with fertilizer use, on maize yields and food security.

## 2 Methodology

### 2.1 Conceptual framework

Improved maize varieties are developed to enhance yields and overcome various stresses that limit productivity (Evenson and Gollin, 2003). Farmers adopt them if their expected utility, through yield and other benefits, is greater than that of local varieties (Jaleta et al., 2018). The adoption of improved maize varieties and varietal replacement is therefore a function of different socio-economic, institutional, and environmental factors (Feder, 1982; Feder et al., 1985). Similarly, farmers replace old varieties when the genetics of the new ones improves their utility, in function of the same factors (Spielman and Smale, 2017).

In the initial phases of agricultural intensification, there is no markets for improved varieties, requiring the public sector to develop them and produce the seed through parastatals (Morris et al., 1998). Such public investments were very successful in the maize sector of East and Southern Africa (ESA) (Smale and Jayne, 2003). However, once farmers start to adopt IMVs, creating a market for seed and other inputs, the private sector is in principle more efficient in producing and disseminating the seed. When the seed sector evolves in its life cycle, the private also starts to take over the developed of new varieties (Morris et al., 1998). In most countries in SSA, agricultural input and output markets were liberalized in the 1990s, including Kenya (Wangia et al., 2004). Understanding the effect of that liberalization on varietal turnover rates, in combination with other factors, is important to plant breeders, informing them on the success of their programs, but also to policy makers by reflecting the success of their liberalization policies, and to researchers to better understand agricultural intensification and the development of the seed industry therein. Finally, understanding the processes in the adoption of improved varieties and varietal change in maize is particularly important for food security in East and Southern Africa, where it is the most important food crop.

Improved varieties and varietal replacement are often associated with increased yields, for example for wheat in Pakistan (Hartell et al., 1998) and in China (Jin et al., 2002), and for maize in Kenya (Mathenge et al, 2014). We hypothesize that varietal turnover increases maize yield, and food production and household food security. Varietal turnover or replacement is the rate at which farmers replace old cultivars, and it is commonly measured by the weighted average age of the current varieties used (Brennan and Byerlee, 1991). Many other factors play a role in yield, especially soil fertility management (Binswanger-Mkhize and Savastano, 2017; Vanlauwe and Giller, 2006), while adoption and varietal turnover are also affected by socioeconomic and institutional factors (Doss et al., 2003).

### 2.2 Farm household survey data

The International Maize and Wheat Improvement Centre (CIMMYT), in collaboration with the Kenya Agriculture and Livestock Research Organization (KALRO) (previously the Kenya Agricultural Research Institute or KARI), conducted four nationally representative household surveys in the major maize growing areas during the last 30 years. The data and the surveys are described elsewhere, as they were used earlier to analyze the trends in mechanization (De Groote et al., 2018) and fertilizer use (Jena et al., 2021).

**Fig. 1:**
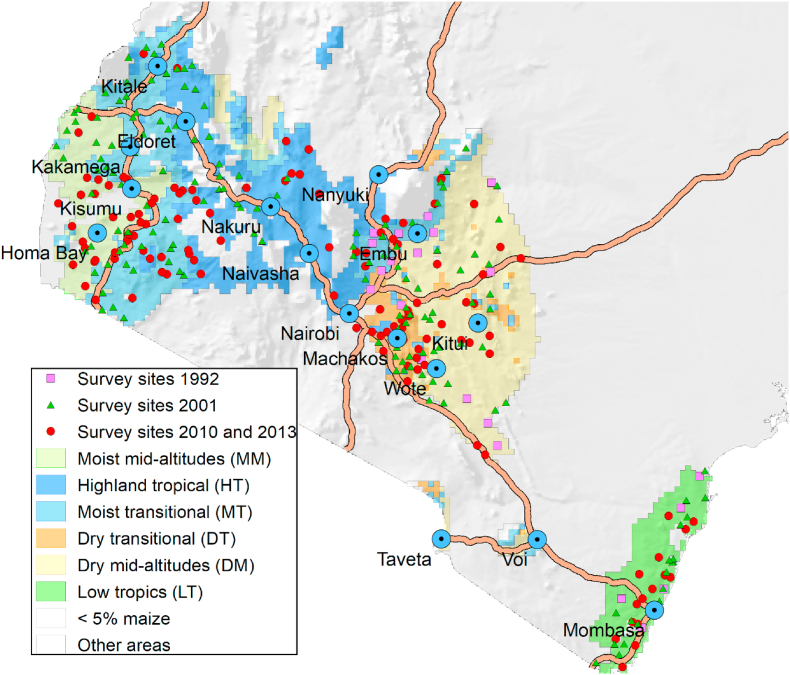
Map of the survey sites, in their respective maize agroecological zones.

All surveys used the same two-stage stratified design with maize production zones or agroecological zones (AEZs) as strata, census clusters or sublocations as primary sampling units, and maize growing households as secondary sampling units. The first survey was conducted in 1992 and covered 79 clusters totaling 1397 farmers (Hassan et al., 1998). This survey also defined the six AEZs, going from the East to West, as the Coastal Lowlands, the Dry Mid-Altitude zone, the Dry Transitional zone, the Moist Transitional zone, the High Tropics and the Moist-Mid Altitude zone (Hassan et al., 1998) (Fig. 1). The second survey, conducted in 2002, covered 185 sublocations based on the 1999 census, with 1652 households (De Groote et al., 2005). The third survey, of 2010, covered 120 sublocations with 1341 households while the fourth survey, of 2012, interviewed the same farmers with a replacement of 20% of randomly sampled households. (Wainaina et al., 2016).

### 2.3 Empirical framework

The key outcome variables in this study are the trends in the adoption of improved varieties, the trends in market share of improved varieties by origin (public versus private sector), the varietal turnover expressed as varietal age, maize yields, and household food security. Adoption of IMVs is expressed as adoption rates or the proportion of farmers adopting, and as adoption intensity or the area share in IMVs, both variables obtained from the surveys. The sample was stratified and representative at the stratum level for each survey year, but national means had to be calculated as weighted means from AEZ means using maize area as weights.

The share of varieties by category was calculated from the surveys for each AEZ and survey year, and at the national level using area weighted means (weights in Supplementary Materials SM1). Varieties were assigned to different categories. The division between public vs. private sector in Kenya is complicated by the evolving role of KSC, initially a private company, later converted to a parastatal, slated for privatization during the liberation of the 1990s, but reestablished as a parastatal in a later reform (Executive office of the President, 2013). At the time of the first survey, 1992, the only IMVs were those developed by KARI and its predecessors and passed on to KSC for seed production. At the time of liberalization, the rights to these varieties were assigned by the court to KSC. We therefore categorize them as “old public varieties”. In the 1980s, KSC had also started developing and marketing their own varieties, independent of KARI, varieties we therefore label “KSC” or parastatal varieties. KARI and its successor KALRO also started developing new varieties independently of KSC but in collaboration with CIMMYT and other public research institutes. These varieties we categorize as “new public varieties”, even when the seed is produced and disseminated by private companies. Varieties developed by the private sector, or whose rights were obtained by the private sector, were now categorized as private sector, distinguishing local and multinational private companies. The list of all improved maize varieties with category and release year is presented in Supplementary Materials 2 (SM2).

Varietal replacement is approximated by the weighted average age of the varieties (*WAA)* (Brennan and Byerlee, 1991). At time *t,* for each variety *i* its share is expressed as *p*_*i*_ and its age as *R*_*it*_ (number of years since its release):

(3) $${WAA}_{t}=\sum_{i}p_{it}R_{it}$$

*WAA* was calculated for individual farmers, for different regions and for the whole country.

The last two surveys were conducted with the same households with 20% replacement; so these data are partially panel data, for which random effects models are indicated (Greene, 2012). For the binary adoption outcome, the probit model was used, for varietal age and food insecurity linear regression.

To analyze the factors that affect adoption of IMVs, a probit model with random effects was used, with socioeconomic, institutional, and geographic variables as factors. Socio-economic factors include characteristics of the household head (such as age, gender and education) and of the household (size, land owned) (Feder et al., 1985). Institutional factors include access to credit and extension services. Previous studies showed that education and access to credit and extensions increase adoption rates of improved varieties or fertilizer (Jaleta et al., 2018; Ouma and De Groote, 2011). Households with larger tracts of land are more likely to adopt improved varieties (Wondale et al., 2016). Geographic and climatic factors include agroecological zone, precipitation, elevation, and are an important determinant of the adoption of technologies such as improved maize varieties (Kaguongo et al., 2012; Njagi et al., 2017).

To analyze the factors that affect varietal turnover, a linear model with random effects was used, with *WAA* as dependent variable and the same factors as in the adoption model as explanatory variables. Previous research showed more experienced farmers continued growing older varieties in Kenya (Smale and Olwande, 2011), while households grow younger varieties when they have more access to extension services (Jamison and Lau, 1982) and have more land (Walker and Alwang, 2015).

To analyze the effect of varietal age on yields, a random effects linear model with yield (kg/ha) as dependent variable was used. In the first model, only *WAA* (in years) was included as exploratory variable*,* in the second model fertilizer use (in kg/ha) and the cross effect with *WAA* were also included. Finally, to analyze the effect of varietal age on food security a random effects linear model was used with household food insecurity access scale (HFIAS) as dependent variable (Coates et al., 2007) and the same explanatory variables as in the adoption model.

## 3 Results

### 3.1 Descriptive statistics of participating farmers

The four surveys show important changes in the household characteristics over time (Table 1). The household head's average age increased, from an average of 49 years in 1992 to 54 in 2013, while education increased from 5 to 8 years, the length of primary schooling in Kenya. The proportion of households with female heads reduced, from 30% to 18%. Household size decreased, from an average of 7 to 6 members. Access to institutional support and infrastructure increased, especially to credit (from 18 to 54%), extension services (41%–85%), and market access (distance to the closest market decreased from 8.7 km to 1.8 km).

**Table 1:** Descriptive statistics of participating farmers in the different survey years.

Group	Variable	1992		2002		2010		2013	
		Mean	Std. Dev	Mean	Std. Dev	Mean	Std. Dev	Mean	Std. Dev
Head	Age of household head (years)	48.74	15.57	47.90	15.11	52.55	15.21	54.42	14.92
	Female (1 = yes, 0 = no)	0.30	0.46	0.26	0.44	0.18	0.38	0.19	0.39
	Household head education (years)	4.81	4.20	4.77	4.24	7.09	4.31	7.72	4.48
Household	Household size (number of members)	7.15	4.37	7.52	4.28	6.12	2.71	6.46	2.56
	Household food insecurity access scale (HHFIAS)					6.82	6.37	6.56	6.60
Institutions	Access to credit (1 = yes:0 = no)	0.18	0.38	0.18	0.39	0.47	0.50	0.54	0.50
	Access to extension services (1 = yes:0 = no)	0.41	0.49	0.35	0.48	0.19	0.40	0.85	0.35
	Distance to the nearest market (km)	8.69	12.78	7.53	7.53	2.04	6.39	1.81	5.89
Farm	Land cultivated (ha)	3.99	10.33	2.71	6.10	1.86	2.25	1.65	2.10
	Cattle ownership (y/n)	0.19	0.40	0.31	0.46	0.64	0.48	0.73	0.44
	Cattle owned (number)	0.54	1.68	0.88	3.87	2.09	3.42	2.70	4.97
	Oxen ownership (y/n)	0.17	0.41	0.20	0.45	0.16	0.36	0.21	0.41
	Oxen ownership (number)	0.23	0.79	0.30	0.91	0.36	1.17	0.57	1.27
Maize	Maize area (ha)	2.61	14.79	1.02	1.75	0.67	0.89	0.85	1.32
	Sale of maize (1 = yes, 0 = no)	0.2	0.40	0.19	0.39	0.25	0.43	0.49	0.50
	Adopts improved maize variety (1 = yes; 0 = no)	0.67	0.46	0.72	0.45	0.68	0.47	0.75	0.43
	Uses commercial fertilizer (1 = yes, 0 = no)	0.52	0.50	0.53	0.50	0.52	0.50	0.60	0.49
	Fertilizer use intensity (kg/ha)	64.26	114.37	73.04	108.8	62.33	109.17	89.08	127.68
	Maize yield (kg/ha)	1498	1484	1192	1217	1093	1021	1288	1452
Maize (weighted means)	Adopts improved maize variety (1 = yes; 0 = no)	0.72	0.46	0.75	0.45	0.71	0.47	0.79	0.43
	Uses commercial fertilizer (1 = yes, 0 = no)	0.62	0.49	0.65	0.48	0.58	0.49	0.65	0.48
	Fertilizer application (kg/ha)	82	117	89	119	68	106	100	136
	Maize yield (kg/ha)	1360	1461	1220	1182	1058	927	1116	1236
	N	1397		1652		1341		1340	

^a^ Weighted with the total maize area of each agroecological zone; SD: Standard deviation.

Farms also changed: farm size decreased from 4 ha to 1.65 ha, and maize area decreased from 2.6 ha to 0.85 ha. The proportion of households engaged in selling maize, however, increased from 20% to 49%. Cattle ownership also increased dramatically, from relatively few (19%) in 1992 to most farmers (72%) in 2013. Oxen ownership, on the other hand, increased only slightly (17–21%).

To follow the trends in input use, we calculated the average adoption of improved maize varieties and fertilizer over time, weighted by maize area for each agroecological zone. The trends in fertilizer use were presented and analyzed in more detail in a previous study (Jena et al., 2020). The results showed that the percentage of farmers who adopted fertilizer increased only slightly, from 62% to 65%, but that the application rate increased more robustly, from 86 kg/ha to 100 kg/ha. Adoption of improved maize varieties also increased slightly, from 72% to 79%. However, despite the increased input use, maize yields did not increase over the study period, in line with the results of other studies.

### 3.2 Trends in the adoption rate and adoption intensity of improved maize varieties (656)

**Fig. 2:**
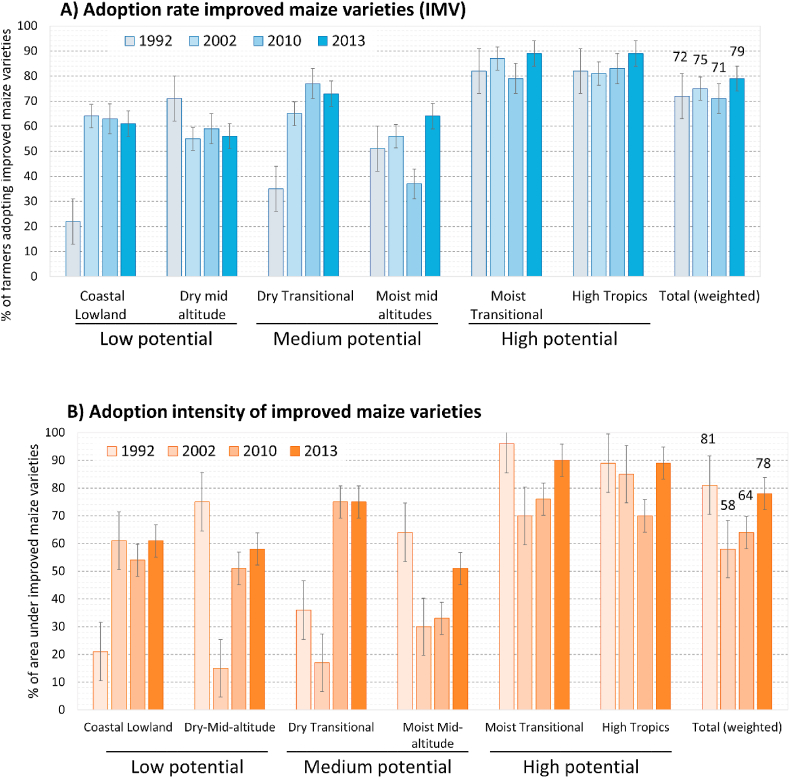
Trends in the adoption of improved maize varieties (IMV) in Kenya from 1992 to 2013, in adoption rate (% of farmers adopting IMVs) and adoption intensity (% of maize area in IMVs) (error bars represent standard errors).

Plotting the average adoption rate of IMVs (weighted by AEZ) over time shows that adoption rates increased only slightly, from 72% to 79% (Fig. 2, Panel A). However, the adoption rates in the first three surveys were nearly the same, so the small increase was only realized between the last two. Adoption rates also differed substantially between the AEZs, with a clear increase in adoption rates along a gradient from low to high potential zones. The highest adoption rates are found in the high potential areas (moist-transitional and high tropics), where almost all farmers (89%) had adopted IMVs by 2013. In the medium-potential areas, the results are lower in the moist mid-altitudes (64%) than in the dry transitional (73%). In the low-potential areas, finally, adoption rates are the lowest, but IMVs are still adopted by more than half the farmers in both the coast (61%) and the dry mid-altitudes (56%).

The adoption trends also varied between the zones, although within zones the differences between years are small in comparison to the standard errors. The high potential areas saw a small increase, especially in the high tropics, but with a dip in the moist transitional zone in 2010. In the medium-potential areas, adoption clearly increased in the dry transitional areas (from 35% to 77% in 2010), while the moist mid-altitudes experienced a gradual decrease over the first three surveys, to increase again in 2013 (to 64% from 37% in 2010). In the low potential areas, the trend was mixed.

Next, the factors affecting the adoption of improved maize varieties were analyzed with a random effects probit model (Table 2). The education of the household head had a positively effect on the adoption of IMVs, but age and gender were not significant. Larger households were also more likely to adopt IMVs. A major factor is market participation: households selling maize are much more likely to grow IMVs. Market participation increased from 20% to 49% over the study period, although that latter level remains low, reflecting the importance of production for home consumption. Among institutional factors, access to extension services increased adoption while distance to the market reduced it, but access to credit was not significant. Adoption also increases with the agricultural potential of the AEZ.

**Table 2:** Determinants of adoption of improved maize varieties (dependent variable binary adoption, using random effects probit regression, marginal effects).

Group	Variable Description	Coefficient	St. Error	p-value	
Household head	Age	−0.003	0.002	0.081	
	Female (1 = yes, 0 = no)	−0.086	0.055	0.117	
	Formal years of schooling of household head	0.029	0.006	0.000	***
Household	Size (number of members)	0.020	0.007	0.003	**
	Total land owned (ha)	0.000	0.000	0.728	
	Household sells maize (1 = yes, 0 = no)	0.140	0.055	0.010	**
Institutional	Access to credit	0.035	0.053	0.509	
	Access to extension services	0.239	0.047	0.000	***
	Distance to the market (km)	−0.005	0.002	0.034	*
AEZ	Dry mid altitude	0.041	0.086	0.638	
	Dry transitional	0.245	0.098	0.012	*
	Moist transitional	0.813	0.088	0.000	***
	High tropics	0.894	0.094	0.000	***
	Moist mid-altitudes	−0.276	0.085	0.001	**
Time	Year of the survey (in years, 1992 = 1)	0.005	0.003	0.148	
Constant	Constant	−0.032	0.118	0.785	
Measures of Fit	Number of Observations	5080			
	Number of groups	4040			
	Rho	0.269			
	Log likelihood	−2460			
	Wald chi2 (16)	303			
	Prob > chi2	0.000			

***p < 0.01, **p < 0.05, *p < 0.1.

Adoption intensity, the area share in IMVs, declined slightly, from 81% to 78% of maize area (Fig. 2, Panel B). However, there was a substantial drop in 2002 (to 58%), followed by slight uptick in 2010 (to 64%) to only surpass the 1992 levels again in 2013. The proportion of area under IMVs also differs across the agroecological zones. As with adoption rates, adoption intensity is higher in the high potential areas, where almost all maize area is in IMVs (90% and 89%). In the medium potential areas, adoption intensity varies from 51% in the moist transitional to 75% in the dry transitional zone. In the low potential zones, finally, adoption intensity averages around 60%. Trends in adoption intensity over time also varied between the zones. Detailed calculations for each variety per AEZ are found in Supplementary Material 3 (SM3).

### 3.3 Sources of maize varieties

**Fig. 3:**
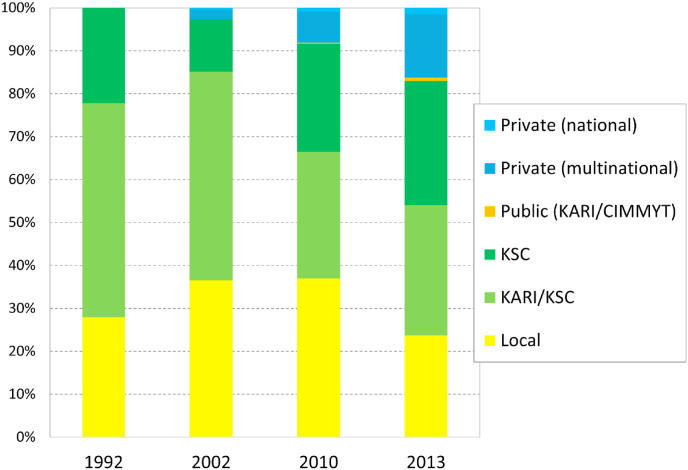
Area share of maize varieties, by source and over time.

To analyze the effect of the liberalization, we calculate the shift from public sector varieties to those from the private sector (Fig. 3). In 1992, before the privatization, the old public KARI/KSC varieties covered half of maize area, while the new (parastatal) KSC varieties covered 22%. This left 28% of maize area in local varieties, while the private sector had not yet come in. After the liberalization in the late 1990s, KARI started developing varieties independently of KSC, and the private sector entered the market. KSC obtained the property rights to the varieties it had been producing while continuing to develop varieties on its own. Still, the proportion of area in IMVs dropped to 66% in 2002 and 62% in 2010, and only increased later (in 2013) to 77%. KSC remained its domination, with more than half of the market share. Progress for the private sector was slow: 2% of the market in 2002 and 16% by 2013. Varieties from the public sector (KARI, its successor KALRO, and CIMMYT), with seed mostly produced and disseminated by local seed companies, covered 6% of the market in 2002 but only reached 9% in 2013.

**Fig. 4:**
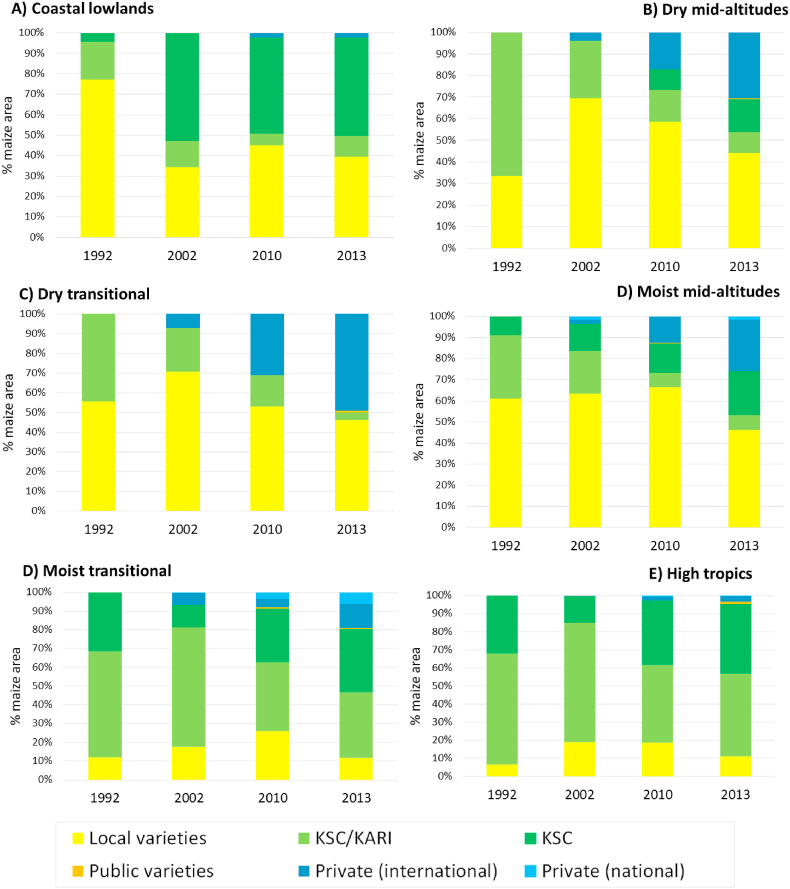
Area share of different types of maize varieties by source and agroecological zone, over time.

Clear differences can be seen between AEZs (Fig. 4). Sorting the zones by their agricultural potential shows a striking resemblance between the lowest potential zone (the coast) and the highest potential (the highlands): in neither zone does the private sector have a significant market share. At the coast, the share of local varieties was reduced but they still covered half the market. The old improved OPVs (coastal composite mostly) were replaced with specific hybrids for the region developed by KSC, while the private sector, without adapted varieties, stayed away. Similarly, the private sector is not active in the highlands, and for similar reasons: it does not have the late-maturing varieties for that zone. The highlands have the highest adoption rate in IMVs, as expected, but also the highest share in old public KARI/KSC varieties, in particular H614 (released in 1986) that still has a remarkable 40% variety share (see SM2 for shares of individual varieties). KSC also made inroads with its new varieties in this zone, but their eight new varieties together still only cover 38% of the market, less than H614 alone. The moist transitional zone, similar to the highlands, is dominated by old public (35%) and new KSC varieties (34%). The private sector did somewhat better here (19%), because of its many medium-maturing varieties suitable for this zone, and the national seed companies also reached their highest market share here (8%).

The private sector made most progress in the drylands, especially in the dry transitional zone (an impressive 58%) but also in the dry mid-altitudes (30%). These varieties are mostly from multinational companies, who have well-adapted and popular varieties. KSC also has replaced most of the old public varieties (drought tolerant OPVs) with new drought tolerant hybrids, except for the old workhorse Katumani Composite (8% in the dry mid-altitudes). In the moist-midaltitude zone, finally, the private sector has also made some progress, with a market share of 26% in the latest survey, and almost all from multinational companies.

### 3.4 Trends in varietal age of maize

**Fig. 5:**
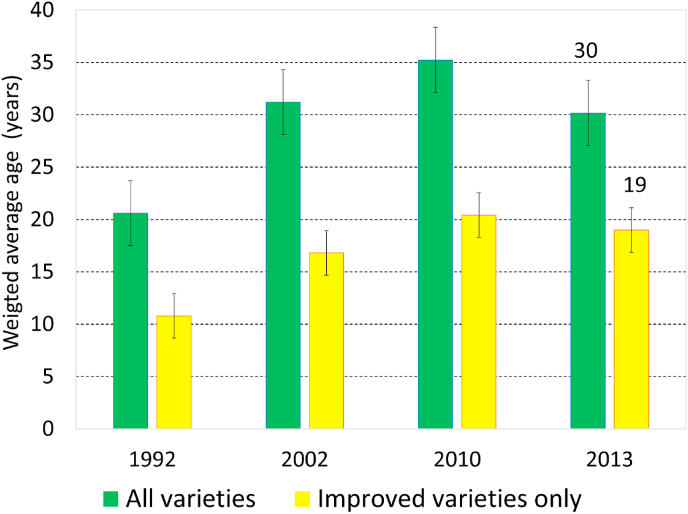
Trends in weighted average age (years) of maize varieties, for all varieties and for improved varieties only.

Next, we calculate the area-weighted average age (WAA) of maize varieties, an inverse indicator of the speed of varietal replacement. In 1992, the WAA was 21 years for all maize varieties and 11 years for improved varieties only, but by 2010, almost 20 years later, both had increased by about 10 years (Fig. 5). The main reasons were the continued popularity of both the old public and local varieties. The WAA only started decreasing between the last two surveys, to 30 for all varieties and to 19 years for improved varieties only, mostly because of the reduction in the share of local varieties and the introduction of new, younger varieties during that period.

**Fig. 6:**
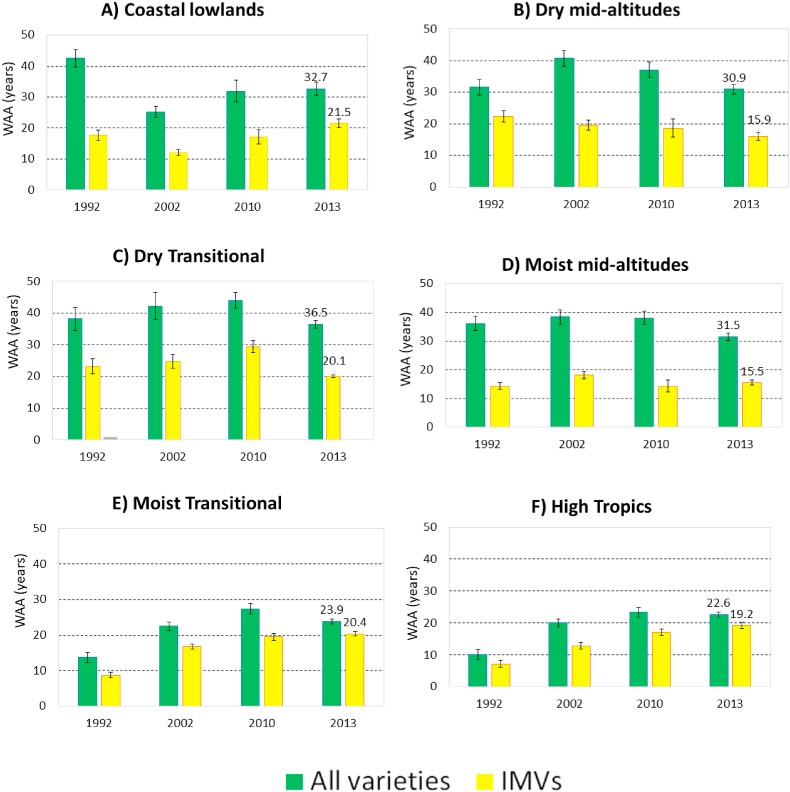
Trends in weighted average age (WAA, in years) of maize varieties, all varieties and improved varieties alone, by agroecological zone.

Varietal age differs substantially across the agroecological zones (Fig. 6). For all varieties, WAA is substantially lower in the high potential areas (23 years in the high tropics and 24 years in the moist transitional), reflecting the lower area share of local varieties, but is much higher in the dry transitional (37 years). For improved varieties only, on the other hand, WAA is substantially lower in the mid-altitudes, both dry (16 years) and moist (15 years), reflecting the progress with improved varieties in general but also with new varieties. Most regions saw a reduction in overall WAA, reflecting some success of new varieties. In the high potential areas, however, WAA increased for all varieties as well as improved varieties only, even over the last two surveys, reflecting the slow progress of varietal replacement there.

Regression was used to analyze the factors affecting varietal age at the household level (Table 3). Households where the head has more education tend to grow younger varieties, while age and gender of the household head were not significant. Larger households tend to grow younger varieties, but larger farmers go more for older varieties. Institutional factors are also important, in particular access to extension, which reduced varietal age by a remarkable 13 years. Access to credit and distance to the market, however, did not affect varietal age. AEZ, finally, also matters, with the high potential zones growing younger varieties.

**Table 3:** Determinants of weighted average age of maize varieties (in years), for the four surveys, using linear random effects.

Group	Variable Description	Coefficient	St. Error	p-value	
Head of household	Age	0.02	0.03	0.451	
	Female (1 = yes, 0 = no)	−1.65	1.04	0.112	‘
	Formal years of schooling	−0.59	0.10	0.000	***
Farm household	Number of members	−0.29	0.12	0.020	*
	Total land owned (acres)	0.01	0.00	0.030	*
	Sale of maize (yes = 1, 0 = no)	−0.70	0.98	0.474	
Institutional/market characteristics	Access to credit	−0.72	0.97	0.457	
	Access to extension services	−13.82	0.83	0.000	***
	Distance to the market in km	0.01	0.05	0.826	
AEZ	Dry mid altitude	17.34	1.80	0.000	***
	Dry Transitional	2.11	1.99	0.290	
	Moist Transitional	−5.64	1.65	0.001	*
	High Tropics	−9.26	1.72	0.000	**
	Moist mid-altitudes	11.53	1.77	0.000	***
Time	Year of the survey (in years, 1992 = 1)	−0.44	0.06	0.000	***
	Constant	52.754	2.287	0.000	***
	Number of Observations	4916			
	Number of groups	3889			
	rho	0.278			
	R^2	0.1633			
	Wald chi2	993			
	Prob > chi2	0.0001			

***p < 0.01, **p < 0.05, *p < 0.1.

### 3.5 Effect of varietal age on yield and food security

The correlation between varietal age and yield was negative and significant for the pooled data, so younger varieties are associated with higher yields (Table 4). The correlation was stronger for all varieties (−0.20) than for just improved varieties (−0.07), likely linked to the arbitrary age of local varieties and the lower yield variance within modern varieties. Similarly, negative and significant correlations between varietal age and yields were found for all individual surveys, except for one, a positive correlation for improved varieties during the last survey (2013), indicating higher yields for the older improved varieties.

**Table 4:** Correlation analysis between maize yield and varietal age (in years), for all varieties and for improved varieties only, between 1992 and 2013 in Kenya.

Year of Survey	All varieties				Improved varieties only			
	Correlation coefficient	p-value	N		Correlation coefficient	p-value	N	
1992	−0.137	0.000	1162	***	−0.089	.010	843	**
2002	−0.232	0.000	1360	***	−0.123	.000	1027	***
2010	−0.320	0.000	1058	***	−0.114	.001	866	***
2013	−0.146	0.000	980	***	0.070	.032	940	*
All	−0.198	0.000	4560	***	−0.072	0.000	3676	***

***p < 0.01, **p < 0.05, *p < 0.1.

This relationship was confirmed by regression analysis (Table 5). In the basic model, with only WAA, yield increases by 7 kg/ha for each varietal age reduction of one year, or an increase of 0.6% over the average yield over the four surveys (1188 kg/ha). In the expanded model, with fertilizer and a general trend, the effect of varietal age is 4 kg/ha/year (an increase of 0.3% over the average), about half the effect without fertilizer, while fertilizer increased yields by 5 kg/ha for each kg (or 0.4%). The cross effect is negative and significant (−0.013), indicating that younger varieties are more responsive to fertilizer. As discussed earlier, weighted average fertilizer application ranged from 82 kg/ha to 100 kg/ha over the study period, with an average of 85 kg/ha over all farmers (N = 5730). However, only 54% of all participants over the four surveys used fertilizers (N = 3114), and their average application rate was 132 kg/ha (unweighted mean by the users). Therefore, the cross effect of varietal age for fertilizer users is a yield increase of 2.5 kg/ha for each year of varietal age. The general trend of yield over time, after taking into account varietal age and fertilizer, is a reduction of 19 kg/ha per year, possibly caused by an expansion of maize area into places less suited and a decrease in soil fertility.

**Table 5:** Effect of weighted average varietal age and fertilizer on maize yield (kg/ha) in four in four household surveys (1992–2013), using random effects model.

	Model 1				Model 2			
	Coeff.	St. error	P		Coeff.	St. errorE	P	
Variety age (years)	−6.63	0.56	0.000	***	−4.16	0.59	0.000	***
Fertilizer (kg/ha)					4.70	0.23	0.000	***
Variety age*fertilizer					−0.01	0.01	0.018	**
Year of survey					−19.11	2.25	0.000	***
Constant	1510	28.1	0.000	***	1342	42.88	0.000	***
No. of observations	5045				5045			
No. of groups	4074				4074			
R-square	0.04				0.19			
Wald chi2 (1)	142				1046			
Rho	0.42				0.37			
Prob > chi2	0.000				0.000			

***p < 0.01, **p < 0.05, *p < 0.1.

Finally, we analyze the effect of varietal turnover on household food security, using HFIAS as an indicator. This score was only collected during the last two surveys and averaged 6.7 (Table 1), on a scale of 0–21. In the short model, with maize technologies only, younger varieties are associated with reduced food insecurity, but the effect is small: for each reduction of age by one year, food insecurity levels reduce by 0.024 or 0.4% (Table 6). The effect of fertilizer is even smaller: 0.007/kg (0.04%), or 0.5 for a 50 kg bag. The long model showed that households food insecurity is lower in households where the head is male and have higher education, households that have more land, have access to credit, are close to the market, and sell maize. Food security is also affected by AEZ, with households located in the high tropics being more and those in the dry mid-altitude zone less food secure.

**Table 6:** Effect of varietal age and other factors on household food insecurity index (HFIAS), for the panel survey data of 2010 and 2013, using the random effects model.

Group	Variable Description	Short model				Long model			
		Coeff.	SE	P		Coeff.	SE	P	
Technologies	Variety age	0.024	0.005	0.000	***	0.013	0.005	0.006	**
	Fertilizer (kg/ha)	−0.007	0.002	0.000	***	−0.003	0.002	0.027	*
	Variety age*fertilizer quantity	0.000	0.000	0.030		0.000	0.000	0.301	
Head	Age of household head					0.000	0.008	0.962	
	Household head is male					0.384	0.329	0.243	
	Education household head (years)					−0.302	0.030	0.000	***
Household	Number of household members					0.286	0.047	0.000	***
	Total land owned (ha)					−0.157	0.030	0.000	***
	Sale of maize (1 = yes, 0 = no)					−1.244	0.252	0.000	***
Institutional factors	Access to credit					−0.818	0.245	0.001	**
	Access to extension services					0.550	0.245	0.025	*
	Distance to the market (km)					0.043	0.020	0.035	*
	Total precipitation (mm/year)					1.094	0.590	0.064	
AEZ	Dry mid altitude					−0.039	0.601	0.948	
	Dry Transitional					−0.865	0.572	0.130	
	Moist Transitional					−2.455	0.593	0.000	***
	High Tropics					−0.296	0.576	0.607	
						8.369	0.840	0.000	***
	Constant	6.581	0.231	0.000	***				***
	Number of Observations	2661				2661			
	Number of groups	896				896			
	R^2	0.0531				0.177			
	Wald chi2	148.97				531			
	Prob > chi2	0.000				0.000			

SE = Standard error.

## 4 Discussion

The analysis of four household representative household surveys conducted over the last 40 years in Kenya shows that varietal age has a significant, but small, positive effect on yield and food security, although the effect on yield is much smaller than that of fertilizer. Unfortunately, the surveys also show low varietal turnover, despite the increased yield of the new varieties and the liberalization of the agricultural sector. While the liberalization led to a large increase in the number of active seed companies and the number of varieties in the market, the parastatal KSC kept its status and most of its market share, especially with its old varieties. Hence, maize yields have not improved over the study period (or thereafter), partly due to slow varietal turnover, but more importantly due to poor soil fertility management. Our analysis shows that the main factors for this lack of progress are stagnation in the adoption of improved varieties, limited success in the liberalization of the seed sector, limited progress in varietal turnover, poor soil fertility management, and limited commercialization of maize.

First, only limited progress was made in the adoption of IMVs: adoption rates were already high in 1992 (72%) and did not increase much (to 79% in 2013). The proportion of the area in improved varieties did not increase, except for the medium-potential zones with substantial area in local varieties. The marginal improvement in adoption of IMVs is similar to that of fertilizer in Kenya, based on analysis of the same survey data (Jena et al., 2021), and to the results from the Tegemeo panel surveys (Smale and Olwande, 2014). Major causes are the already high levels of adoption, and the limited yield increase of improved varieties in marginal areas. Other factors that affect adoption, from our results, are education of the household head, household size, market participation, access to extension and markets, and the agricultural potential of the location. Similar factors were also shown in other studies in Kenya (Ouma and De Groote, 2011) and Ethiopia (Jaleta et al., 2018), while a study based on the Tegemeo data also found a strong positive effect of the grain/price ratio on adoption of hybrids (Smale and Olwande, 2014). There is a clear difference in adoption of new varieties between the agroecological zone, and their success in stress environments is a clear result of targeted breeding for those environments and the introduction of adapted material from the private sector, especially multinationals.

The second major factor of stagnation is the limited effect of the privatization. Adoption of varieties from the private sector remains limited, and the domination of KSC persists, with both old and newer varieties. Private companies made some progress in the dry transitional and mid-altitudes, but not in the high potential areas, especially the highlands, as it has few suitable varieties for those. This dominance has been observed in the past (Nambiro et al., 2001) and in more recent studies, based on national data (Nagarajan et al., 2019) and the Tegemeo surveys (Smale and Olwande, 2014). The main causes of factors are strong political support for the parastatal, the low price it offers compared to private seed companies, and a uniform price irrespective of package size, type of variety (hybrid vs. OPV) and zone (De Groote et al., 2005; Swanckaert et al., 2011). Further, KSC, unlike the private sector, has developed hybrids suitable for the lowland tropics and, most importantly, has late-maturing varieties very suited for the highlands, unlike the private sector.

The third factor is the low varietal turnover in maize, as indicated by high varietal age. WAA actually increased over the study period with the exception of the last survey, to 30 years for all varieties, which is similar to that of a previous study: 30 years for Kenya and 31 years for East and Southern Africa (Walker and Alwang, 2015). Our estimate of WAA for improved varieties only is 19 years, higher than the previous estimate of 15 years for ESA (Walker and Alwang, 2015), but in line with the 2009 Tegemeo survey data, 18.3 years (Smale and Olwande, 2014). A study in drought-prone areas, on the other hand, estimated it at 13 years (Abate et al. (2017). Our study further shows that age of improved varieties decreases with education, family size and access to extension, and with high potential areas, but increases with farm size. Households with higher education levels and more access to extension are more likely to have more access to information about newer varieties and their benefits (Naseem et al., 2018; Walker and Alwang, 2015). We found an effect of market participation on adoption, but not on varietal age, unlike another study (Spielman and Smale, 2017). Our findings on land size is in contrast with the Tegemeo results, where larger commercially oriented farmers grew younger varieties (Smale and Olwande, 2014). New varieties also have to competing with older released varieties (Walker and Alwang, 2015), especially when production is mainly for subsistence (Spielman and Smale, 2017).

When farmers grow younger varieties, however, yields increase, as shown from the strong negative correlation between varietal age and yield. The effect is, however, small, an increase of 4.16 kg/ha/year, similar to the effect of 1 kg of fertilizer: 4.7 kg/ha. A positive cross effect of varietal age and fertilizer use indicates that younger varieties respond better to fertilizer. The effect of varietal age on yield is in line with the Tegemeo results (Smale and Olwande, 2014), but in contrast with a more recent study at the national level where the number of varieties and area in IMVs did not increase yield (Nagarajan et al., 2019). The main causes of low varietal turnover can be summarized as the persistent dominance of KSC, the strong performance of their old varieties in contrast to the limited yield gains from young varieties, risk-aversion of farmers to new varieties in contrast to familiarity with the old ones, and low market penetration of the private sector, especially in the high-potential areas (for which it does not have suitable late-maturing varieties).

The fourth factor in the low performance of the maize sector is soil fertility. Our results show that there is only a slight increase of fertilizer adoption over time (from 62 to 65%), and is positively affected by education, farm size and agricultural potential. On the positive side, the application rate is increasing more strongly (from 86 to 100 kg/ha. Other studies have shown that soil fertility varies strongly with intensification (Tittonell et al., 2005), and that poor soil fertility management is related to farmers’ lack of knowledge, variable response across the soil fertility gradient and high cost (Misiko et al., 2011). The last factor, low market participation, is affected by the still high proportion of people living in rural areas (72% in 2021), resulting in high level of self-sufficiency for basic staples, low returns to maize as compared to vegetables and fruits and, especially, dairy (Thorpe et al., 2000). Small-scale farmers also face difficulties accessing maize markets, although this has improved substantially over time with improved roads and mobile phones (Mather et al., 2013).

Finally, results show that varietal age (and fertilizer) reduce food insecurity, although the effect of both agricultural practices is small. We also found food insecurity to be decreasing in function of age and education of the household head, land size, maize sales, access to credit and markets. extension services. The factors with the highest effect on food security were selling maize and access to credit.

Our study faced several limitations. First, pooled data sets from different surveys were used, and only the last two rounds were panel data. Second, to examine the effect of varietal turnover on food security we only used the last two rounds, which only cover a short time period. Third, the last survey was from 2013, so new trends might have escaped the analysis. Fourth, the studies did not take into account several factors, in particular soil fertility and the price of inputs like seed and fertilizer. Still, the survey data cover a long period of time, and, unlike other data sets, have a clear sampling design and are representative of the different maize production zones of the country.

We conclude that young maize varieties, the product of recent maize breeding efforts, do have a significant but small effect on yield and food security in Kenya. Unfortunately, this has not enticed farmers to adopt younger varieties to a large scale, despite the agricultural liberalization which allowed the entry of many private seed companies and a large number of new varieties with increased yield and stress resistance, as KSC keeps dominating the market. In combination with a stagnating use of fertilizer (Jena et al., 2021), this led to stagnating yields during the study period and beyond.

Based on our result, policy recommendations include those that level the field for the private sector to compete with the parastatal KSC. Further, as soil fertility at this stage in agricultural intensification is the major factor in maize yields, it should receive more attention from research and extension. To improve the adoption of IMVs, finally, recommendations based on our results include participatory variety evaluation to ensure the varieties fit the farmers’ needs. Access to extension also needs to be promoted, both through the public extension service and the private sector, which now dominates seed dissemination, to ensure farmers chose the right varieties for their situation. Policies should also promote ensure universal education, especially in rural areas. Given its important effect on the adoption of IMVs, farmers need to be encouraged and supported to participate in the market, by promoting good drying and storage practices, and increasing access to markets, in particular by providing market information and infrastructure and reducing transaction costs.

## Declaration of competing interest

The authors declare that they have no known competing financial interests or personal relationships that could have appeared to influence the work reported in this paper.

## Data Availability

The data will be curated and published with the questionnaires on the CIMMYT website. https://hdl.handle.net/11529/10548862

## Appendix A Supplementary data

Supplementary Materials with this paper include the weights for the maize areas and population in the different AEZs and surveys (SM1), a list of all varieties with their origin and date of release (SM2) and the area share of each variety in each AEZ in each survey (SM3). By the time of publication, the household survey data used for this paper will be published on the CIMMYT website, with the questionnaires.
