# Supplementary material for: Varietal turn-over and their effect on yield and food security – Evidence from 20 years of household surveys in Kenya
Source: Glob Food Sec. 2023 Mar;36:100676. doi: 10.1016/j.gfs.2023.100676 (PMC10015270; doi:10.1016/j.gfs.2023.100676)
Supplement: Multimedia component 1 [file mmc1.pdf]

Supplementary Material 1. Improved maize varieties in Kenya

Source: National variety lists, consulted in 2013 and 2021

Variety Name	Variety Type	Year Released	Origin of Variety	Type of Organization Releasing Variety	Company producing	Type of Organization Producing Variety
H631	Hybrid	1964	KARI	Public	Kenya Seed Company	Public
H622	Hybrid	1965	Kenya Seed Company	Public	Kenya Seed Company	Public
H632	Hybrid	1964	KARI	Public	Kenya Seed Company	Public
H512	Hybrid	1967	Kenya Seed Company/ KARI	Public	Kenya Seed Company	Public
Katumani Composite	OPV	1967	Kenya Seed Company/ KARI	Public	Kenya Seed Company	Public
H513	Hybrid	1970	Kenya Seed Company/ KARI	Public	Kenya Seed Company	Public
Coast Composite	OPV	1974	Kenya Seed Company/ KARI	Public	Kenya Seed Company	Public
H625	Hybrid	1981	KARI/ Kenya Seed Company	Public	Kenya Seed Company	Public
DLC_Makueni	OPV	1986	KARI	Public	Kenya Seed Company	Public
H511	Hybrid	1967	Kenya Seed Company/ KARI	Public	Kenya Seed Company	Public
H613	Hybrid	1986	KARI/ Kenya Seed Company	Public	Kenya Seed Company	Public
H614	Hybrid	1986	KARI/ Kenya Seed Company	Public	Kenya Seed Company	Public
H626	Hybrid	1989	KARI/ Kenya Seed Company	Public	Kenya Seed Company	Public
PH1	Hybrid	1989	Kenya Seed Company	Public	Kenya Seed Company	Public
DHO1	Hybrid	1995	Kenya Seed Company	Public	Kenya Seed Company	Public
DHO2	Hybrid	1995	Kenya Seed Company	Public	Kenya Seed Company	Public
H515	Hybrid	1995	Kenya Seed Company/ KARI	Public	Kenya Seed Company	Public

Variety Name	Variety Type	Year Released	Origin of Variety	Type of Organization Releasing Variety	Company producing	Type of company producing
H627	Hybrid	1995	KARI/ Kenya Seed Company	Public	Kenya Seed Company	Public
Pan5195	Hybrid	1995	Pannar Seed Company	Private	Pannar Seed Company	Private
PH4	Hybrid	1995	Kenya Seed Company	Public	Kenya Seed Company	Public
PH3253	Hybrid	1996	Pioneer Hybrid	Private	Pioneer Hybrid	Private
H623	Hybrid	1999	Kenya Seed Company	Public	Kenya Seed Company	Public
H628	Hybrid	1999	Kenya Seed Company	Public	Kenya Seed Company	Public
CG4141	Hybrid	2000	Cargill	Private	Monsanto	Private
DHO3	Hybrid	2000	Kenya Seed Company	Public	Kenya Seed Company	Public
H516	Hybrid	2000	Kenya Seed Company/ KARI	Public	Kenya Seed Company	Public
H629	Hybrid	2000	Kenya Seed Company	Public	Kenya Seed Company	Public
Pan5355	Hybrid	2000	Pannar Seed Company	Private	Pannar Seed Company	Private
DHO4	Hybrid	2001	Kenya Seed Company	Public	Kenya Seed Company	Public
FaidaSeed650	Hybrid	2001	OCD (Faida Seeds)	Private	OCD (Faida Seeds)	Private
H520	Hybrid	2001	Kenya Seed Company/ KARI	Public	Kenya Seed Company	Public
H6210	Hybrid	2001	Kenya Seed Company	Public	Kenya Seed Company	Public
H6212	Hybrid	2001	Kenya Seed Company	Public	Kenya Seed Company	Public
Kh600 15A	Hybrid	2001	CIMMYT/KARI	Public	East African Seed	Public
Kh600 16A	Hybrid	2001	CIMMYT/KARI	Public	Freshco	Public
Pan67	Hybrid	2001	Pannar Seed Company	Private	Pannar Seed Company	Private
Pan691	Hybrid	2001	Pannar Seed Company	Private	Pannar Seed Company	Private
H6213	Hybrid	2002	Kenya Seed Company	Public	Kenya Seed Company	Public

Variety Name	Variety Type	Year Released	Origin of Variety	Organ. Releasing	Organization Producing	Type company
MasenoDC	OPV	2002	Lagrotech Seed Company	Private	Lagrotech Seed Company	Private
Pioneer	Hybrid	2002	Pioneer Hybrid	Private	Pioneer Hybrid	Private
DK8031	Hybrid	2003	Monsanto	Private	Monsanto	Private
DK8071	Hybrid	2003	Monsanto	Private	Monsanto	Private
H611	Hybrid	2003	KARI/ Kenya Seed Company	Public	Kenya Seed Company	Public
WS403	OPV	2003	CIMMYT	Public	Western Seed Company	Private
WS502	OPV	2003	CIMMYT	Public	Western Seed Company	Private
WS505	OPV	2003	CIMMYT	Public	Western Seed Company	Private
DK8053	Hybrid	2004	Monsanto	Private	Monsanto	Private
H6214	Hybrid	2004	Kenya Seed Company	Public	Kenya Seed Company	Public
H624	Hybrid	2004	Kenya Seed Company	Public	Kenya Seed Company	Public
Kakamega Synthetic	OPV	2004	KARI	Public	Kenya Seed Company	Public
Kh500 21A	Hybrid	2004	KARI	Public	Dryland Seed Limited	Private
SCDuma41	Hybrid	2004	Agri Seed Company Ltd.	Private	Agri Seed Company Ltd.	Private
SCDUMA43	Hybrid	2004	Agri Seed Company Ltd.	Private	Agri Seed Company Ltd.	Private
Simba	OPV	2005	Agri Seed Company Ltd.	Private	Agri Seed Company Ltd.	Private
Kdv 1	OPV	2006	CIMMYT	Public	Dryland Seed Limited	Private
Kdv 6	OPV	2006	CIMMYT	Public	Dryland Seed Limited	Private
H612	Hybrid	2008	KARI/ Kenya Seed Company	Public	Kenya Seed Company	Public
PAN7M 97	Hybrid	2008	Pannar Seed Company	Private	Pannar Seed Company	Private
Panam 419	Hybrid	2008	Pannar Seed Company	Private	Pannar Seed Company	Private
WS105	OPV	2008	CIMMYT	Public	Western Seed Company	Private
