# Supplementary material for: Varietal turn-over and their effect on yield and food security – Evidence from 20 years of household surveys in Kenya
Source: Glob Food Sec. 2023 Mar;36:100676. doi: 10.1016/j.gfs.2023.100676 (PMC10015270; doi:10.1016/j.gfs.2023.100676)
Supplement: Multimedia component 2 [file mmc2.pdf]

Supplementary Material 2. Maize agroecological zones in Kenya

Maize agroecological zones in Kenya, with estimated maize area and production in 1992, 2005 and 2010

Agroecological zone	Elevation	Maize statistics (household survey 1992)			SPAM 2005			SPAM 2010			Population (WorldPop)	Weights			
		Area (1000 ha)	Production (1000 tonnes)	Yield (t/ha)	Area (1000 ha)	Production (1000 tonnes)	Yield (t/ha)	Area (1000 ha)	Production (1000 tonnes)	Yield (t/ha)	(1000)	Area	Production	Production (6 zones)	Population
Lowland Tropics	0-700	41	53	1.29	47	37	0.77	56	30	0.54	2,857	0.03	0.01	0.02	0.06
Dry Mid-altitude	700-1400	166	162	0.98	320	170	0.53	386	196	0.51	3,825	0.19	0.08	0.10	0.08
Dry-Transitional	1100-1700	66	76	1.15	79	35	0.44	566	486	0.86	5,403	0.28	0.20	0.25	0.12
Moist-transitional	1200-2000	466	1234	2.65	499	1,219	2.44	372	524	1.41	7,931	0.19	0.22	0.27	0.17
Highlands	1600-2900	316	909	2.88	347	810	2.33	239	586	2.45	1,801	0.12	0.25	0.30	0.04
Moist Mid-altitude	1110-1500	173	231	1.34	173	357	2.06	99	109	1.10	12,137	0.05	0.05	0.06	0.26
Maize production zones								1,717	1,932	1.12				1.00	
< 5%					67	130	1.94	88	119	1.57	1,858	0.04	0.05		0.04
Other					141	169	1.20	202	326	0.86	10,076	0.10	0.14		0.22
Total		1244	2671	2.1471	1,674	2,927	1.75	2,007	2,376	1.18	45,890	1.00	1.00		1.00
