# Supplementary material for: Varietal turn-over and their effect on yield and food security – Evidence from 20 years of household surveys in Kenya
Source: Glob Food Sec. 2023 Mar;36:100676. doi: 10.1016/j.gfs.2023.100676 (PMC10015270; doi:10.1016/j.gfs.2023.100676)
Supplement: Multimedia component 3 [file mmc3.pdf]

Supplementary Material 3

List improved maize varieties and area share (%), by year and zone, category and company

1.Coastal lowlands

Variety name	Category	Company	1992	2001	2010	2013
Coast Composite	KARI/KSC	KSC	6.9	8.1		4.5
DHO1	KSC	KSC				1.1
DHO2	KSC	KSC				2.2
DHO4	KSC	KSC			1.1	1.1
H520	KSC	KSC				1.1
Kakamega Synthetic	KSC/KARI	KSC			0.8	
Katumani Composite	KARI/KSC	KSC	11.5	4.6	4.9	5.6
PAN7M 97	Private (intern.)	PANNAR			2.3	
Makueni Composite - DLC1	KSC/KARI	KSC		0.1		
PH1	KSC	KSC	4.4	16.3	13.5	9
PH4	KSC	KSC		36.5	32.5	33.7
SCDUMA43	Private (intern.)	SeedCo				2.2
Improved varieties			22.8	65.6	54	60.5
Local varieties			77.2	34.4	46	39.5

2. Dry Mid-altititudes

Variety name	Category	Company	Area share of variety (in %)			
			1992	2001	2010	2013
CG4141	Private (int)	Cargill		0.3	0.2	
Coast Composite	KSC/KARI	KSC			0.1	
DHO1	KSC	KSC			2.7	3.2
DHO2	KSC	KSC			3.3	6.5
DHO4	KSC	KSC			3.4	5.1
DK8071	Private (intern.)	Cargill			0.9	1.4
DK8071	Private (intern.)	Cargill				0.4
H511	KSC/KARI	KSC	7.4	2.1	0.2	0.5
H520	KSC/KARI	KSC				0.5
H512	KSC/KARI	KSC	4.4			
H613	KSC/KARI	KSC	1.2			
H614	KSC/KARI	KSC	1.9			
H625	KARI/KSC	KSC	1.4			
H626	KSC/KARI	KSC	1.2		0.5	
Katumani Composite	KSC/KARI	KSC	48.8	14.9	13.4	8.3
Kdv 1	CIMMYT/KARI	Dryland seeds				0.5
Makueni Composite - DLC1	KSC/KARI	KSC	0.3	9.7	0.6	0.5
Pan67	Private (intern.)	PANNAR			0.4	0.9
PH1	KSC	KSC			0.1	0.4
PH3253	Private (intern.)	Pioneer		3.6		3.2
PH4	KSC	KSC			0.1	
Pioneer	Private (intern.)	Pioneer			0.1	
SCDUMA41	Private (intern.)	SeedCo			0.9	1.4
SCDUMA43	Private (intern.)	SeedCo			14.5	23.1
Improved varieties			66.6	30.6	41.4	55.9
Improved varieties			33.4	69.4	58.6	44.1

3. Dry Transitional Zone

Variety name	Category	Company	Area share of variety (in %)			
			1992	2001	2010	2013
CG4141	Private (intern.)	Cargill		1.1		
DHO1	KSC	KSC			2.4	0.5
DHO2	KSC	KSC			1.1	3.4
DHO3	KSC	KSC				0.5
DHO4	KSC	KSC			3.2	1.5
DK8031	Private (intern.)	Cargill				12.8
DK8053	Private (intern.)	Cargill				0.1
DK8071	Private (intern.)	Cargill			1.7	0.5
H511	KSC/KARI	KSC	33.1	3.7	1.5	
H512	KSC/KARI	KSC	8.7			
H513	KSC	KSC		1.6	1	3
H520	KSC	KSC				2
H6210	KSC	KSC			0.5	
H6213	KSC	KSC			0.3	
H614	KSC/KARI	KSC	0.5			
H625	KSC/KARI	KSC	1.9	0.3	0.1	
H627	KSC/KARI	KSC		0.4		
H628	KSC	KSC		0.4		
Katumani Composite	KSC/KARI	KSC		16	12.3	2
Kdv 1	CIMMYT/KARI	Dryland seeds				1
Makueni Composite - DLC1	KSC/KARI	?		1.2	0.3	2.5
Pan5195	Private (intern.)	PANNAR			0.4	
Pan67	Private (intern.)	PANNAR			0.8	1
Pan691	Private (intern.)	PANNAR				0.5
PAN7M 97	Private (intern.)	PANNAR			0.5	0.5
PH1	KSC	KSC			1.1	
PH3253	Private (intern.)	Pioneer		6		11.3
PH4	KSC	KSC			0.8	0.5
SCDuma41	Private (intern.)	SeedCo				1.5
SCDUMA43	Private (intern.)	SeedCo			24.2	30
Simba	Private (intern.)	SeedCo			0.1	
Improved varieties			44.2	30.7	52.3	75.1
Local varieties			55.8	69.3	47.7	24.9

4. Moist Transitional

Variety name	Category	Company	Area share of variety (in %)			
			1992	2001	2010	2013
CG4141	Private (intern.)	Cargill		0.9		
Coast Composite	KARI/KSC	KSC	0.6			
DHO3	KSC	KSC			0.2	
DHO4	KSC	KSC			0.2	1.1
DK8030	Private (intern.)	Cargill			0.1	
DK8031	Private (intern.)	Cargill				3.7
DK8053	Private (intern.)	Cargill				0.8
DK8071	Private (intern.)	Cargill			1.1	0.3
H511	KSC/KARI	KSC	5.7	6.9	0.6	0.3
H512	KSC/KARI	KSC	4.8	2	0.4	0.3
H513	KSC	KSC		2.9	8.1	9
H515	KSC	KSC				0.6
H516	KSC	KSC			1	2
H520	KSC	KSC			0.3	1.1
H524	KSC	KSC				0.3
H611	KARI/KSC	KSC			0.2	
H612	KARI/KSC	KSC			0.1	
H613	KARI/KSC	KSC	0.6		1.4	1.1
H614	KARI/KSC	KSC	43.3	52.9	31.7	31
H615 (not in NVL)	KARI/KSC	KSC			1.1	
H616 (not in NVL)	KARI/KSC	KSC			0.3	
H6210 (N)	KSC	KSC			3.1	1.7
H6212	KSC	KSC				0.3
H6213	KSC	KSC			2.5	5.1
H6214	KSC	KSC				0.3
H622	KSC/KARI	KSC	1.2	0.2		
H623	KARI/KSC	KSC			0.1	
H624	KSC	KSC			3	4.2
H625	KSC	KSC	22.4	6.3	4.3	3.7
H626	KSC	KSC	9	0.2	1	0.3
H627	KSC/KARI	KSC		1.6		
H628	KSC	KSC		1.8	2.8	2
H629	KSC	KSC		0.1	1.6	1.5
H632	KARI/KSC	KSC	0.2			
Katumani Composite	KSC/KARI	KSC		0.3	0.8	0.8
Kh500 21A	KARI	Freshco seed (?)			0.1	
Kh600 15A	KARI	Freshco seed (?)				0.3
Kh600 16A	KARI	Freshco seed (?)			0.8	0.3
Kitale Synthetic (N)	KARI/KSC	KSC	0.1			
Makueni Composite	KARI/KSC	KSC				1.4
MasenoDC	Lagrotech seed co.	Lagrotech seed co.		0.2		
Pan5195	Private (intern.)	PANNAR			0.4	

Pan67	Private (intern.)	PANNAR			0.1	0.3
Pan691	Private (intern.)	PANNAR			0.3	0.2
Pan419	Private (intern.)	PANNAR				0.3
PAN7M 97	Private (intern.)	PANNAR			0.3	
PH1	KSC	KSC		0.6		
PH3253	Private (intern.)	Pioneer		5.7		2.8
PH4	KSC	KSC			0.5	0.6
SCDUMA43	Private (intern.)	Seedco			2	4
Simba	Private (intern.)	Seedco				0.3
WS403	Private (local)	Western Seed Co			0.3	0.8
WS502	Private (local)	Western Seed Co			0.3	0.3
WS505	Private (local)	Western Seed Co			3	5.1
Improved varieties			11.1	0.9	68	88.2
Local varieties			88.9	99.1	32	11.8

5. High Tropics

Variety name	Category	Company	Area share of variety (in %)			
			1992	2001	2010	2013
Coast Composite	KARI/KSC	KSC	0.1		0.4	
DHO4	KSC	KSC			0.1	0.4
DK8031	Private (intern.)	Cargill				0.8
FS650	Private (local)	Faida Seeds			0.3	
H511	KSC/KARI	KSC	4.9	0.6	1	
H512	KSC/KARI	KSC	2.3		0.4	
H513	KSC	KSC			0.9	0.8
H520	KSC	KSC				1.3
H611	KSC	KSC	0.3		0.2	
H613	KARI/KSC	KSC	3.3		0.9	1.3
H614	KARI/KSC	KSC	50.3	57.6	37.7	40.3
H6210 (N)	KSC	KSC			5.2	0.8
H6213	KSC	KSC			5.5	16.4
H622	KSC/KARI	KSC		0.3	0.3	
H624	KSC	KSC			10.5	6.3
H625	KSC	KSC	18.8	1.8	0.4	0.4
H626	KSC	KSC	12.8	3.2	3.6	0.8
H627	KSC/KARI	KSC		6.9	0.7	1.7
H628	KSC	KSC		9.9	7.9	4.6
H629	KSC	KSC			1.4	6.7
Katumani Composite	KSC/KARI	KSC	0.6	0.3	1.3	2.5
Kh500 21A	KARI	Freshco seed (?)			0.1	
Kh600 15A	KARI	ADC				1.3
Pan5195	Private (intern.)	PANNAR			0.2	
Pan5355	Private (intern.)	PANNAR			0.2	
Pan67	Private (intern.)	PANNAR			0.1	
Pan691	Private (intern.)	PANNAR			0.7	0.8
PAN7M 97	Private (intern.)	PANNAR			0.5	
PH3253	Private (intern.)	Pioneer		0.3		
PH4	KSC	KSC			0.1	
SCDUMA43	Private (intern.)	Seedco			0.4	1.3
WS403	Private (local)	Western Seed Co			0.2	0.4
Improved varieties			93.4	80.9	81.2	88.9
Local varieties			6.6	19.1	18.8	11.1

6. Moist Mid-Altitudes

Variety name	Category	Company	Area share of variety (in %)			
			1992	2001	2010	2013
DHO1	KSC	KSC			0.4	0.8
DHO4	KSC	KSC			3.9	2.1
DK8031	Private (intern.)	Cargill				3.8
DK8071	Private (intern.)	Cargill			0.1	0.8
H511	H511	KSC	10	4.4	1.8	1.7
H512	H512	KSC	4.9	0.4	0.8	0.3
H513	H513	KSC		3.4	4	5
H516	KSC	KSC			0.3	1.3
H520	KSC	KSC	0			2.5
H611	KSC	KSC			0.2	
H613	KARI/KSC	KSC	0.6			0.8
H614	KARI/KSC	KSC	12.5	14	1.6	4.2
H6210 (N)	KSC	KSC			0.4	0.4
H6213	KSC	KSC				0.1
H624	KSC	KSC		2.6	1.3	0.4
H625	KSC	KSC	7.6	5.5	1.1	1.3
H626	KSC	KSC	1.3		0.4	0.5
H627	KSC/KARI	KSC			0.2	
H628	KSC	KSC				0.4
H632	KARI/KSC	KSC	1.8			
Katumani Comp	KSC/KARI	KSC	0.2	1.5	0.4	
Kh500 21A	KARI	PANNAR			0.1	
Makueni Compo	KSC/KARI	KSC			1.9	
MasenoDC	Lagrotech seed co.	Lagrotech seed co.		1.5	0.4	1.5
PAN	Private (intern.)	PANNAR		0.2		
Pan67	Private (intern.)	PANNAR				0.8
Pan691	Private (intern.)	PANNAR			0.1	
PH1	KSC	KSC		1.3	1.9	2.5
PH3253	Private (intern.)	Pioneer		1.8		0.4
PH4	KSC	KSC			0.1	3.3
SCDUMA43	Private (intern.)	SeedCo			1.2	4.6
Simba	Private (intern.)	SeedCo			1.8	2.1
WS105	Private (intern.)	Western Seed Co			0.4	
WS403	Private (intern.)	Western Seed Co			0.6	5
WS505	Private (intern.)	Western Seed Co			8	7.1
Improved varieties			38.9	36.6	33.4	53.7
Local varieties			61.1	63.4	66.6	46.3
